# Cincho-Quinine

**Published:** 1875-04

**Authors:** 


A CARD.—We desire to call the attention of the faculty to the following analyses of Cincho-Quinine, from some of the most eminent chemists. BILLINGS, CLAPP & Co., *Boston.*

CHEMICAL LABORATORY OF THE UNIVERSITY OF PENNSYLVANIA.
WEST PHILADELPHIA, January 29, 1875.

*Messrs. Billings, Clapp, & Co.:*

GENTLEMEN: I have received by express a package marked "Sealed by S. P. Sharples, January 22, 1875," and containing a bottle of Cincho-Quinine, with the label of James R. Nichols & Co., Chemists, Boston, which I have tested, and have found it to contain *quinine, quinidine, cinchonine,* and *chinchonidine.*

Yours respectfully,
F. A. GENTH,
*Professor of Chemistry and Mineralogy.*

LABORATORY OF THE UNIVERSITY OF CHICAGO.
CHICAGO, February 1, 1875.

I hereby certify that I have made a chemical examination of the contents of a bottle of Cincho-Quinine, and by direction I made a qualitative examination for *quinine, quinidine* and *cinchonine,* and hereby certify that I found these alkaloids in Cincho-Quinine. C. GILBERT WHEELER,
*Professor of Chemistry.*

